# Evaluating the effect of maternal non-communicable disease on adverse pregnancy outcomes and birthweight in Pakistan, a facility based retrospective cohort study

**DOI:** 10.1038/s41598-023-51122-z

**Published:** 2024-01-05

**Authors:** Esther Wainwright, Irfan Sheikh, Rahat Qureshi, Sana Yousuf, Raheela Khan, Matthew Elmes

**Affiliations:** 1 Division of Food, Nutrition and Dietetics, School of Biosciences, University of Nottingham, Loughborough, LE12 5RD UK; 2 Aga Khan University Hospital, Stadium Road, Karachi, 74800 Pakistan; 3 School of Medicine, Royal Derby Hospital Centre, University of Nottingham, Translational Medical Sciences Unit, Derby, DE22 3DT UK

**Keywords:** Diseases, Reproductive disorders

## Abstract

Non-communicable diseases (NCDs) claim 74% of global lives, disproportionately affecting lower and middle-income countries like Pakistan. NCDs may increase the risk of preterm birth (PTB), caesarean section (CS), and low birthweight. This study aims to determine whether the high prevalence of NCDs in Pakistan play a role in the high rates of preterm births, and CS. This retrospective cohort study from Aga Khan University Hospital, Pakistan, investigated effects of pre-existing NCDs on pregnancy outcomes of 817 pregnant women. Medical records were used to generate odds ratios for the risk of PTB, labour outcome and birthweight in women with type 1 and type 2 diabetes, hypertension, asthma and thyroid disorders. Multinomial logistic regression and general linear models were used to adjust for confounding variables using IBM SPSS Statistics (v27). Type 2 diabetes significantly increased the risk of PTB and elective CS (both *P* < 0.05). Elective CS was significantly increased by hypertension and asthma (both, *P* < 0.05). Surprisingly, asthma halved the risk of PTB (*P* < 0.05), while type 1 diabetes significantly increased birthweight from 2832 to 3253g (*P* < 0.001). In conclusion, pre-existing NCDs increase the risk of negative pregnancy outcomes, including PTB, elective CS and birthweight. Asthma, however reduced PTB and justifies further investigation.

## Background

Poor birth outcomes, including preterm birth (PTB) and low birth weight (LBW) are a major public health problem across the world, particularly in lower- and middle-income countries (LMICs) such as Pakistan. Central and southern Asia account for 17% of all maternal deaths worldwide with maternal mortality rates for southern Asia estimated to be 134 per 100,000 live births^1^. Complications of pregnancy and childbirth remains the leading cause of death and disability for childbearing women in Pakistan^2^. Preterm birth (born before 37 weeks' gestation) is the second leading cause of death under 2 years of age worldwide and is a major concern to global policy makers^3^. PTBs disproportionately affect LMICs with more than 60% occurring in Africa and South Asia^4^. Rates in Pakistan exceed 15%, making them the top 10 highest globally for PTB^4^. LBW (< 2500g) rates are found to be 12.6% in Pakistan and seen as one of the highest LBW rates globally^5^. Both PTB and LBW increase the risk of the offspring developing non-communicable diseases including hypertension, heart disease, and type 2 diabetes in their adult lives^6^.

Despite caesarean sections improving maternal and foetal outcomes where necessary, the World Health Organization (WHO) has stated that there is no additional benefits when CS rates increase above 10–15%^7^. A recent study estimated CS rates in Southern Asia to be 19%^8^, with a reported 19.6% CS rate in 2018 for Pakistan, far exceeding the WHO recommendations^9^. CS can affect current and future pregnancies as well as having implications for the offspring. Several cohort studies have found an increased risk of severe acute maternal morbidity, including haemorrhage, hysterectomy, uterine rupture, and obstetric shock^10^. Subsequent pregnancy following a CS is also associated with an increased risk of stillbirth, premature delivery, uterine rupture and abnormal placentation^10^. Offspring born via CS compared to those delivered vaginally also face significantly different short term and long-term health outcomes. Short-term risks include altered immune development, allergy, asthma and reduced gut microbiome diversity^10^. In later adult life, those born via CS are at greater risk of developing adiposity, hypertension, altered liver function, neurological and stress related problems^10^.

South-Asia also has some of the highest rates of NCDs globally and often described as an epidemic^6^. Within Pakistan, studies have found a high burden of NCDs in urban^11^ and semi-urban settings^12^. In the South Asian mega city, Karachi, 8% of the population are diabetic and 18% hypertensive. Furthermore, 39% of the population have been determined to be pre-hypertensive and 40% pre-diabetic, emphasising that the burden will only increase further^11^. This widespread occurrence of NCDs not only affects older^12^ generations, but also spans the entire population including women of reproductive age^13^. A study with a specific focus on women from the Bhimber District in the north-east of Pakistan found that a total of 6% of girls and women within the population have a NCD with diabetes, high blood pressure and asthma being in the top 10 most common NCDs identified^14^.

Not only do adverse birth outcomes put the adult offspring at greater risk of developing NCDs but there is some evidence mothers with pre-existing NCDs are at greater risk of delivering premature and LBW babies^3,6^. This creates an intergenerational cycle causing negative health impacts throughout the population. A recent scientometric analysis of births in Pakistan identified priorities’ to address their poor maternal and infant health outcomes. The suggested outcome was that focus should be on identifying the key factors affecting maternal and infant morbidity but also the early life causes and predictors of NCDs to understand the impact, but also to identify new ways to address the problem^6^. Currently, Pakistan is not on track to meet the recommended improvements to reduce maternal and infant morbidity and mortality^6^. Due to population growth and limited resources, the situation seems to be worsening. Maternal health is the key to improving wellbeing today and for the future^13^. A full understanding of the drivers of adverse pregnancy outcomes are required to help to break the intergenerational cycles and improve the health of the Pakistan population. With the combined high burden of NCDs and adverse pregnancy outcome rates that Pakistan currently faces and very little research investigating the effect of a mother exhibiting multiple NCDs on birth outcomes, an overview of the problem is required. The current study used logistic regression to explore the effect of the common NCDs^11^, pre-gestational diabetes Type 1 and Type 2, hypertension, asthma and thyroid disorder on adverse pregnancy outcomes in a cohort of Pakistani women. This study will give us a clearer idea of why premature birth and CS rates are so high within Pakistan but also what influence NCD’s have on the risk. The hypothesis the study aims to investigate is that NCDs will increase the risk of adverse pregnancy outcomes.

## Methods

### Participants and data

The data used in the analysis is from a retrospective cohort study that took place at Aga Khan University Hospital, Pakistan. This study was performed in accordance with the relevant guidelines and regulations from the Institutional Ethics Review Committee (IERC) and involved analysis of pre-existing secondary data from participants providing informed consent for its use and did not involve any direct participant interaction. All participant data was anonymised and analysed in an aggregated form to ensure the privacy of the participants involved. Therefore, the research was exempted from ethical approval by the IERC at Aga Khan University. The cohort consists of pregnant women, admitted to the obstetrics department that gave birth between 2015 and 2016. The data collected comprised of maternal age, marital status and socioeconomic status. Participant body mass index (BMI) was calculated using height and weight records prior to pregnancy. A detailed medical history including details of their NCDs, the duration of these diseases and obstetric information from previous pregnancies were retrieved (live births, miscarriages, ectopic pregnancies, induced abortions, caesarean sections, stillbirths). For the current pregnancy very detailed obstetrical information was also recorded (gestational age at delivery, labour and delivery types, delivery outcome, medical and obstetric complications, infections, ICU/critical care admissions, duration of hospital stays, birthweight, APGAR scores, gender, birth injuries and any other anomalies). Women were included in the study if they had at least one of the following pre-existing NCDs: diabetes Type 1 and Type 2, hypertension, asthma and thyroid disorder. 857 records were available. All of those with missing information were excluded leaving, 817 subjects that were included in the final analysis. Non-communicable disease burden was characterised by summing the number of diseases an individual had.

### Variables

The primary aim of this study was to look at the effect of NCDs on pregnancy outcomes, so premature birth (before 37 weeks/37 + weeks gestational age at delivery), type of labour (spontaneous, elective CS, emergency CS or Induction) and birth weight (g) were chosen as the outcome variables. The following confounding variables were chosen based on existing knowledge of an interaction with our outcome variables, these included maternal age (years); BMI (kg/m^2^); gravida (number of live births + number of still births), CS and preterm births; pre-eclampsia (yes/no) ; sex of offspring (male/female); birth weight (g) and gestational age at delivery (weeks—not used in preterm).

### Statistical analysis

All statistics were done in IBM SPSS Statistics program V.27 and figures produced in GraphPad Prism 7. Frequency tables, percentages and means were utilised to present demographic information on previous and current birth data. For categorical variables, crosstabs were produced indicating the frequency and proportion of women with each NCD for all pregnancy outcomes. Crude odds ratios (cOR) were calculated for premature birth and type of labour (with spontaneous birth used as the reference category). Chi-squared tests were used to determine if there was a significant association. Multinomial logistic regression models were then run to produce the adjusted ORs (aOR) including all NCDs and confounding variables for preterm birth and type of labour outcomes. Means and standard deviations were calculated, and independent sample t-tests were run for birth weight and each NCD group. A general linear model including all NCDs and confounding variables was run for birthweight to adjust for these confounders.

### Ethics approval and consent to participate

As the study involved human subjects not involved directly, and with no intervention the Institutional Ethics Review Committee at Aga Khan University issued and approved an exemption letter for full ethics review prior to the study commencing.

## Results

### Demographics

A total of 817 women delivered 817 singleton babies over the 2 year period. Demographics and previous birth data are shown in Table 1. The vast majority of women were married, housewives aged between 28 and 36, classified as obese from the South Asian BMI classifications. Geographical location was used to determine socioeconomic status and 99.8% of the women were determined to have satisfactory status. A satisfactory socioeconomic status was classified as individuals being able to afford to pay for their care in the private not-for-profit institution and being of upper or middle-income status living in affluent areas of the city and its outskirts.

**Table 1:** Demographic information, previous birth history for all 817 women included in the analysis.

Information on mothers		F (%)
Age (mean =30.8)	37+	100 (12.2%)
	28–36	505 (61.9%)
	< 27	212 (25.9%)
Marital status	Divorced	5 (0.6%)
	Married	812 (99.4%)
Occupation	Employed	64 (7.8%)
	Housewife	753 (92.2%)
Socioeconomic status	Satisfactory	815 (99.8%)
	Not Satisfactory	2 (0.2%)
BMI (mean = 28.6)	Underweight	22 (2.7%)
	Normal	102 (12.5%)
	Overweight	248 (30.4%)
	Obese	445 (54.5%)
Previous live births history		
Live Births	No live births	279 (34.1%)
	One live birth	251 (30.7%)
	Two live births	153 (18.7%)
	Three live births	91 (11.1%)
	Four or more live births	44 (5.3%)
Miscarriages	No previous miscarriages	558 (68.3%)
	One previous miscarriage	156 (19.1%)
	Two previous miscarriages	61 (7.5%)
	Three or more previous miscarriages	42 (5.2%)
Ectopic pregnancies	No ectopic pregnancies	804 (98.4%)
	One or more ectopic pregnancies	13 (1.6%)
Stillbirths	No previous stillbirths	784 (96%)
	One previous stillbirth	27 (3.3%)
	Two or more previous stillbirths	6 (0.7%)
Induced abortions	No induced abortions	796 (97.4%)
	One or more induced abortions	21 (2.5%)
Caesarean Sections	No previous CS	493 (60.3%)
	One previous CS	176 (21.5%)
	Two previous CS	92 (11.3%)
	3 or more previous CS	56 (6.8%)
Preterm	No previous PTB	737 (90.2%)
	One previous PTB	68 (7.1%)
	Two or more previous PTB	22 (2.7%)

Count (F) and percentage of all women shown for each category and mean for maternal age, BMI.

The prevalence of different non-communicable health conditions among the surveyed population is outlined in Table 2. Type 1 and 2 diabetes was present in 2.4% and 11.6% of individuals respectively. Hypertension was prevalent in 18.7% of admitted patients, whereas asthma and thyroid disorders affected 33.4% and 46% of the population. Regarding the overall burden of NCDs, 88.4% of admitted obstetric patients had one NCD, whereas 11.7% were observed to make up individuals who had two or three NCDs.

**Table 2:** Non-communicable disease prevalence and current birth data for all 817 women included in the analysis.

NCDs		Number of women (%)
Diabetes type 1	Yes	20 (2.4%)
	No	797 (97.6%)
Diabetes type 2	Yes	95 (11.6%)
	No	722 (88.4%)
Hypertension	Yes	153 (18.7%)
	No	664 (81.3%)
Asthma	Yes	273 (33.4%)
	No	544 (66.6%)
Thyroid disorder	Yes	376 (46%)
	No	441 (54%)
Burden	One NCD	722 (88.4%)
	Two NCDs	83 (10.2%)
	Three NCDs	12 (1.5%)
Current birth data		
Sex of offspring	Female	397 (48.6%)
	Male	420 (51.4%)
Gestational age at delivery (Mean = 37)	Less than 28	4 (0.6%)
	28–31	13 (1.6%)
	32–36	183 (22.5%)
	37+	616 (75.3%)
Pre-eclampsia	Yes	36 (4.4%)
	No	78 (95.6%)

NCD burden was calculated by how many NCDs each woman had. Count and percentage of all women shown for each category and mean calculated for gestational age at delivery.

The current birth data is also shown in Table 2. Sex of the offspring, were equally matched with admitted patients giving birth to 48.6% female and 51.4% male offspring. A number of babies were born prematurely, 0.6% were born before 28 weeks gestation, whereas, 1.6% born between 28 and 31 weeks gestation and a further 22.5% between 32 and 36 weeks of age. The majority of babies were born at 37 weeks or later (75.3%) and pre-eclampsia was reported in 4.4% of all pregnancies.

### Preterm labour

**Figure 1:**
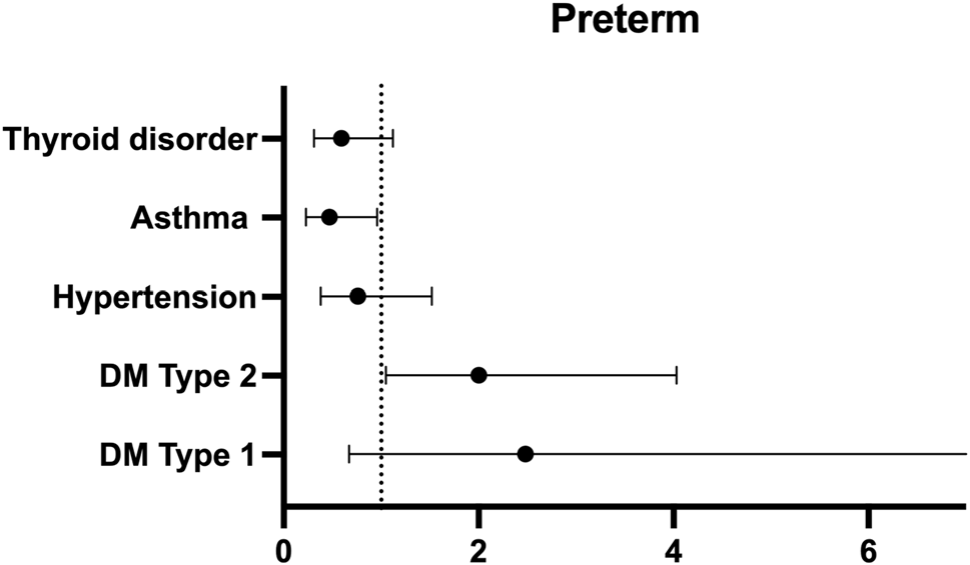
aOR and 95% CI for preterm birth from the final logistic regression model including all NCDs and confounding variables. Dotted line = 1. Any crossing the line are not considered significant. Diabetes type 2 was found to significantly increase the odds of preterm birth (aOR = 2.05, *p *= 0.04) and Asthma to decrease (aOR = 0.49, *P *= 0.047).

Of the 817 women included in the study, 201 women (24.6%) delivered before 37 weeks gestational age so were classified as preterm. The proportion of term and preterm births for each disease is shown in Table 3. Both type 2 diabetes and hypertension significantly increased the risk of preterm birth (Table 3). The presence type 2 diabetes increased the crude odds ratio (cOR) of delivering preterm by 2.5 fold and hypertension 1.5 fold respectively. However, asthma was found to significantly reduce the crude risk of delivering preterm, nearly halving the odds of delivering preterm if the mother was asthmatic with a cOR of 0.58 (Table 3). Interestingly, neither thyroid disorder nor type 1 diabetes had a significant effect on preterm birth. The final logistic regression model including all NCDs and accounting for confounding variables identified that the adjusted odds ratio (aOR) for type 2 diabetes still remained significant increasing the odds of delivering prematurely twofold (aOR = 2.07, *P* < 0.05). Hypertension however was no longer significant after confounding variables were considered (Fig. 1). Interestingly the protective effect of asthma also remained significant halving the odds of a preterm birth (aOR = 0.49, *P* < 0.05), (Fig. 1).

**Table 3:** Showing the proportion of those who delivered term and preterm for each disease group and the crude odds ratios, which does not account for confounding variables.

	Preterm	Term	cOR	*P* value
Diabetes type 1				
Yes	4 (20%)	16 (80%)	1.31	0.629
No	197 (24.7%)	600 (75.3%)		
Diabetes type 2				
Yes	40 (42.1%)	55 (57.9%)	2.52	*P* < 0.001
No	161 (22.3%)	561 (77.7%)		
Hypertension				
Yes	49 (32%)	104 (68%)	1.58	*P* < 0.05
No	152 (22.9%)	512 (77.1%)		
Asthma				
Yes	50 (18.3%)	223 (81.7%)	0.58	*P* < 0.01
No	151 (27.8%)	393 (72.2%)		
Thyroid disorder				
Yes	89 (23.7%)	287 (76.3%)	1.09	0.568
No	112 (25.4%)	329 (74.6%)

### Type of labour

**Figure 2:**
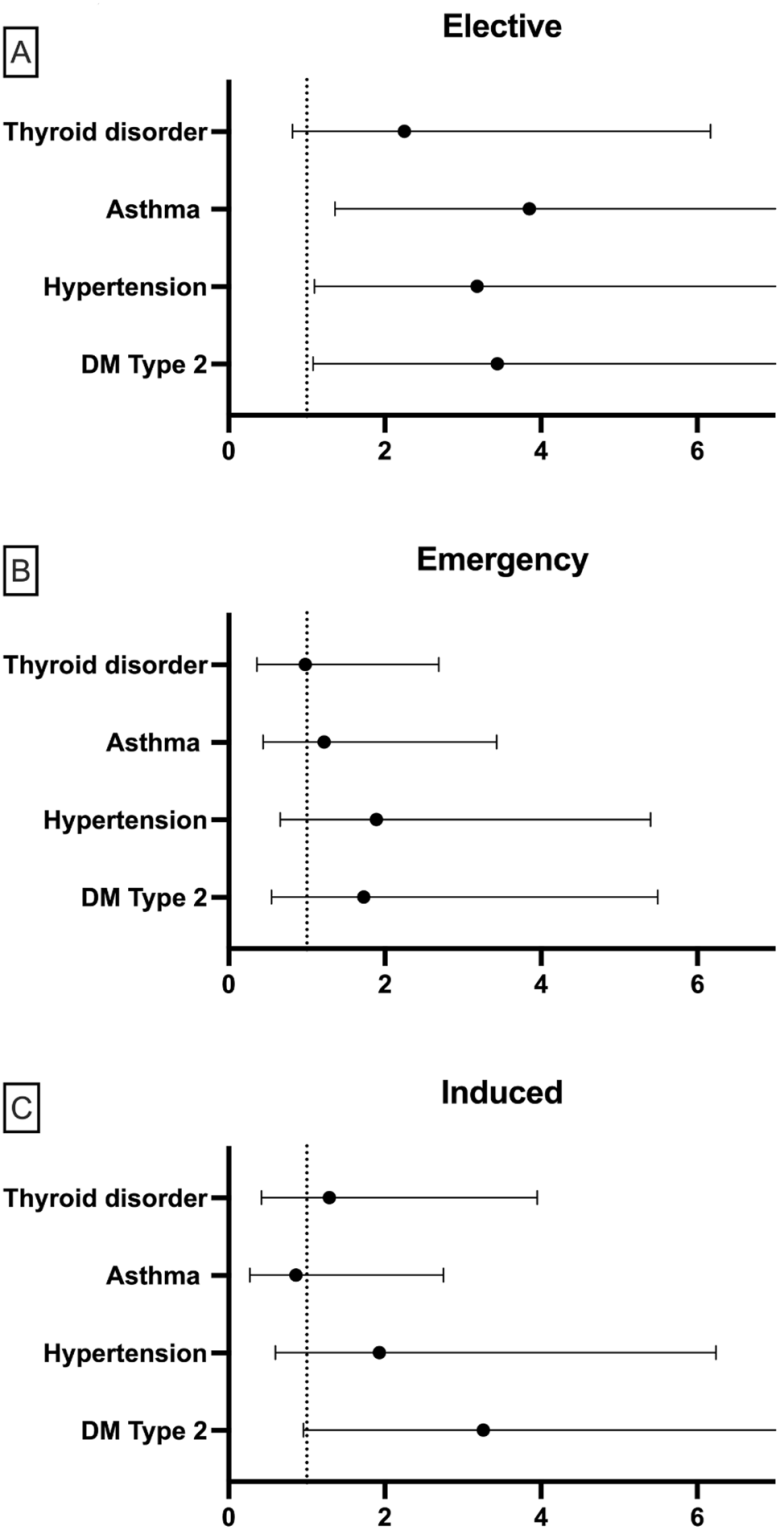
Adjusted odds ratio (aOR) and 95% CI for each type of labour (**A**) Elective CS, (**B**) Emergency CS and (**C**) Induced birth compared to spontaneous birth from the final logistic regression model including all NCDs and confounding variables. Dotted line = 1. Any that cross the dotted line are not considered significant. Diabetes type 2, Asthma and hypertension all significantly increase the odds of elective CS.

Of the 817 women in the study, 163 (19.9%) had a spontaneous birth, 281 (34.4%) had an elective CS, 269 (32.9%) had an emergency CS and 104 (12.7%) were induced (see Table 4). Type 1 diabetes could not be included in the model as the low numbers created unexpected singularities in the hessian matrix and so all those with type 1 diabetes were excluded from this analysis. Type 2 diabetes was found to have the greatest significant effect on type of labour increasing the odds by over twofold for elective CS, emergency CS and induced birth compared to spontaneous birth (Table 4). Hypertension also increased the risk of CS births by over twofold (Table 4). However, asthma decreased the risk of emergency CS and labour induction (Table 4). In contrast, thyroid disorder had no significant effect on the type of labour participants experienced. After adjusting for confounding variables type 2 diabetes (aOR = 3.56 *P* < 0.05), hypertension (aOR = 3.14, *P* < 0.05) and asthma (aOR = 3.89, *P* < 0.05) were all found to significantly increase the odds of elective CS by over threefold (Fig. 2a), however, labour induction and CS were unaffected by the different NCDs (Fig. 2b,c).

**Table 4:** Showing the count and proportion for each type of labour and the crude odds ratios (cOR) without accounting for confounding factors.

NCD		Type of labour									
		Spontaneous	Elective CS			Emergency CS			Induction		
		Count (proportion)	Count (proportion)	cOR	*P* value	Count (proportion)	cOR	*P* value	Count (proportion)	cOR	*P* value
Diabetes Type 1	Yes	0 (0%)	7 (35%)			12 (60%)			1 (5%)		
	No	163 (20.4%)	274 (34.3%)			257 (32.2%)			103 (12.9%)		
Diabetes Type 2	Yes	9 (9.5%)	40 (42.1%)	2.84	*P *< 0.01	33 (34.7%)	2.38	*P *< 0.05	13 (13.7%)	2.44	*P *< 0.05
	No	154 (21.3%)	241 (33.4%)			236 (32.7%)			91 (12.6%)		
Hypertension	Yes	17 (11.1%)	57 (37.3%)	2.19	*P *< 0.01	60 (39.2%)	2.47	*P *< 0.01	19 (12.4%)	1.92	0.071
	No	146 (22%)	224 (33.7%)			209 (31.5%)			85 (12.8%)		
Asthma	Yes	62 (22.7%)	107 (3.2%)	1.01	0.993	77 (28.2%)	0.65	*P *< 0.05	27 (9.9%)	0.57	*P *< 0.05
	No	101 (18.5%)	174 (32%)			192 (35.3%)			77 (14.1%)		
Thyroid disorder	Yes	81 (21.5%)	121 (32.2%)	0.74	0.134	121 (32.2%)	0.82	0.342	55 (14.6%)	1.13	0.611
	No	82 (18.6%)	162 (36.7%)			148 (33.6%)			49 (11.1%)		

Type 1 diabetes was excluded from analysis due to the low numbers preventing the model to determine statistical significance.

### Birthweight

Mean birthweight was 2839g with 211 (25.5%) classed as having a low birthweight (2500g and below) and 10 (1.2%) as macrocosmic (above 4000g). Women with type 1 diabetes exhibited a significantly higher mean birthweight of 3254 ± 472g compared to 2833 ± 588g (*P* = 0.002). This remained significant after adjusting for confounding factors and all other NCDs in a generalised linear model (F = 14.77 *P* < 0.001) (Table 5). Birthweight was not significantly affected by any other NCD.

**Table 5:** Showing the mean birthweight ± standard deviation (g) for each disease group.

		Birthweight (g) Mean +/−SD	T-test	GLMF	*P* value
Diabetes Type 1	Yes	3254 ± 472	***P* = 0.002**	**14.77**	***P* < 0.001**
	No	2833 ± 588			
Diabetes Type 2	Yes	2783 ± 676	*P* = 0.294	1.06	0.3
	No	2858 ± 572			
Hypertension	Yes	2776 ± 655	*P* = 0.12	1.75	0.19
	No	2858 ± 572			
Asthma	Yes	2871 ± 552	*P* = 0.33	1.49	0.22
	No	2829 ± 606			
Thyroid disorder	Yes	2822 ± 563	*P* = 0.35	2.59	0.11
	No	2860 ± 610			

Independent T tests were performed for each group to determine significant differences. A Generalised linear model was performed including all NCDs and confounding variables. Those in bold remained significant.

## Discussion

NCDs have been shown on multiple occasions to have negative implications on birth and pregnancy outcomes^1,15–17^. This study provides evidence that NCDs increase the risk of some negative outcomes including preterm birth, elective CS and induced birth but also that asthma is associated with a significant decrease in the risk of PTB.

Nearly a quarter (24.7%) of the births in this study were premature (before 37 weeks) which fits the current landscape, with Pakistan being identified as one of the top 10 countries in the world for high PTB rates^18^. Recent research identified PTB rates to be approximately 22% for the whole country and when broken down by province found rates of 22.5% for the Sindh province, which is where the hospital providing data for the current study is located^19^. Only type 2 diabetes was found to significantly increase the risk of PTB. Studies based in Japan, Spain and the UK corroborate this by also finding type 2 diabetes to increase risk of preterm birth however these also found type 1 to increase the risk also findings^20–23^. A linear relationship between fasting plasma glucose and negative pregnancy outcomes (including PTB) has been shown in China^23^. However, it is also worth noting that type 2 diabetes also increased the crude risk ratio for elective CS and induced birth by over threefold (cOR = 3.44 and cOR = 3.26 respectively). This is not surprising as clinical management of diabetes in pregnant mothers is suggested to induce labour at 40 + 6 weeks when no other complications are present but with metabolic problems it is suggested to deliver at 37–38 weeks gestation. This is further encouraged due to Pakistan being a resource poor setting and to avoid unexplained uterine death, it’s advised to not exceed 40 weeks, even with women with controlled diabetes^24^. Of the 40 women who had type 2 diabetes and delivered preterm, 13 had elective CS and 2 were induced. 21 of the women had an emergency CS. Even in countries such as Australia where prenatal care is readily available, glycaemic control is well managed and with specialist care, type 2 diabetes remains a risk for several negative pregnancy outcomes^16^. Soholm et al. 2021 found pre-eclampsia and foetal asphyxia to be the main reasons for delivering preterm in diabetic pregnancies^25^.

One of the most important and interesting findings of the current study is that asthma was associated with a decreased risk of PTB by over half with an aOR of 0.47. This contrasts with the published literature, where evidence suggests there is no significant association with maternal asthma and preterm birth^26^. However, it was found to decrease preterm risk when asthma was managed properly^27^. Management and control of asthma during pregnancy seems to be an extremely important factor in decreasing the risk of adverse pregnancy outcomes. Well controlled asthma has been proven to reduce exacerbations during pregnancy, improving maternal and foetal outcomes^28^. Where asthma was found to increase the risk of preterm birth, it was evident that mothers treated for asthma had a reduced risk of delivering prematurely compared to those untreated^29^. The type of medication used to treat asthma has been found to significantly effect PTB extremes, Oral corticosteroids have been linked to preterm birth < 32 weeks, short-acting Beta-adrenergic-agonists to births < 37 weeks and < 32 weeks respectively but inhaled corticosteroids associated with a reduced PTB rate^30^.

Poorly managed asthma can lead to foetal hypoxia and stress leading to release of cortisol releasing hormone and inflammatory cytokines, which can trigger PTB. Protection against this with well-managed asthma and inhaled corticosteroids, in particular, suggests a protection against inflammation and asthma^30^. The current study had no information on the type of medication pregnant women with asthma were receiving so this could not be accounted for in the analysis. It is important to note that there are many factors that impact on PTB ranging from socioeconomic status to obstetrical and gynaecological history with complex interactions of mother, foetus and environment that are all indirect and so can be difficult to unravel^31,32^. Low maternal weight, previous preterm deliveries, anaemia and physical and emotional stress are factors that have been identified to be associated with the risk of preterm birth in Pakistan^33^. Epidemiological factors such as young maternal age, older paternal age and shorter pregnancy intervals have been identified as risk factors for PTB, along with environmental factors such as infections, excessive alcohol use and smoking^32^. Nutrition and diet are also likely to have a big impact particularly as research provides evidence that a diet higher in omega 6 PUFA increases the risk of PTB^34^.

Those with type 1 diabetes were found to have a significantly higher birthweight than those without. Previous studies have also found type 1 diabetes to increase birthweight to the greatest extent in comparison to type 2 and gestational, relative to pregnancies without diabetes^35^. This has been shown to be influenced by both glycaemic status and placental health, with suboptimal glucose levels and healthy placentas producing the heaviest babies^36^ and by maternal BMI^37^. Potential factors related to this include hyperinsulinemia in the fetus (induced by fetal hyperglycaemia or maternal hyperglycaemia), glycaemic control, reduced plasma ghrelin levels and some conflicting evidence that leptin levels are involved^38^. Despite 25.5% of the women in this study delivering LBW offspring, none of the NCDs looked at exhibited any significant associations with lower birthweights. Another study based in Pakistan found significant variation of LBW incidence within specific sociodemographic groups including maternal health indicators, pregnancy history and geo-demographic factors^39^.

It was also evident from the current study that nearly all of the NCDs investigated increased the risk of elective CS. Several other studies are in agreement where hypertension^40–42^, diabetes^43,44^, thyroid issues^15^ and asthma^1,27^ were found to increase operative deliveries. Similar studies also based in Pakistan have found the same NCDs to be risk factors for increased CS but often these are grouped under the term “pregnancy complications” and included diabetes, hypertension, respiratory disorders and thyroid problems that were also grouped with other issues such as cardiac problems and anaemia^9,45^. Interestingly there was no significant association found with emergency CS or the NCDs within the current study, however, it is key to note that the previously mentioned studies did not differentiate between elective and emergency CS so it is difficult to make precise comparison.

A final yet very important finding of the current study is the large number of CS being carried out. Over half the women gave birth via an operative delivery, at 67.5% with elective CS (34.3%) being the most popular type of delivery, followed closely by emergency CS (33.2%). These rates vary hugely in different studies all within Pakistan–ranging from as low as 14%^9^ up to 21.4%^46^, 34%^41^ and 69.7%^45^. This variation in CS rate depends on the characteristics of the region or hospital each study was carried out. Many studies have found higher socioeconomic standing, more urban areas^45^ and private hospitals to be significant risk factors for CS^9,47^. In other countries such as India the same trend persists^48^. It is also worth noting the rates here may be inflated due to all the women having an NCD so could impact clinical decisions. Regardless, the rates in this study far exceed the maximum 15% recommended by the World Health Organization. There are several hypothesised drivers of high CS rates including private health care and general lack of education and opinion on the risks associated with CS. To try and unravel the reasons behind high CS rates, one study via a questionnaire asked women about their knowledge and influences for why they may choose to have a CS. All women said they would have a CS if their physician recommended it^45^. Reasons for mothers favouring CS over other types of birth without medical indication have been documented as being a fear of labour pain, pelvic floor damage and repercussions and it’s often perceived in these women to be safer for them and the baby as it can be portrayed as easier and more convenient. For healthcare practitioners, a fear of being sued for malpractice and to a much lesser extent convenience and planning are drivers behind agreeing to an elective. In many private health care settings, there are increased numbers of CS compared to the public sector if CS can generate more revenue for the hospital. Furthermore, in resource poor settings, lack of skilled professionals has been attributed to the high rates of CS in tertiary care hospitals^49^.

Antenatal care is also an important factor in looking at the drivers behind adverse pregnancy outcomes. Studies have found that a lack of ante-natal care to be a risk factor for PTB^50^ and to be important for identifying the risk of PTB and influencing factors^32^. The WHO recommends at least 4 ante-natal visits and less than this has been found to significantly increase risks of CS in Pakistan^9^. This could be due to the fact that conditions such as NCDs can be captured and managed effectively with appropriate advice form physicians and so improve birth outcomes and decrease the need for operative deliveries. For example, identifying exacerbations of diseases, the onset of gestational conditions such as pregnancy-induced hypertension and proper management of existing conditions like asthma^27^ are all important for reducing poor paediatric outcomes. Unfortunately, we didn’t have any information provided on ante-natal care of women involved in the study, but it would have been an interesting variable to include.

This study has limitations. The data collected was from only sampling from one geographical area and hospital, potentially reducing the generalisability of the study. Pakistan has a privatised health care system and CS rates can often be higher. However, Aga Kahn University hospital is a non-profit hospital that has 4 secondary care facilities in Karachi and Hyderabad which receive a large number of referrals from urban and rural areas of the Sindh and other provinces and referrals for specialised treatment could potentially cause the high number of operative deliveries. Finally, all the women selected for the study had a previous NCD recorded so there was no control group for comparison. This was adjusted accordingly in the final logistic regression models by accounting for all NCDs as each NCD was included. The retrospective nature of the study meant several women had missing information and the data cannot determine cause and effect.

In conclusion, this study is unique in the fact that it includes a range of NCDs separately within our logistic regression model, which to our knowledge has not been done before. Overall, NCDs did effect pregnancy outcomes within this cohort in both negative and positive ways. Type 2 diabetes was found to increase the risk of PTB, whereas asthma was associated with a decreased risk of PTB. Three out of the five NCDs increased the risk of elective CS, however, emergency CS was not affected by any of the NCDs. Thyroid disorder was not found to be associated with any outcomes investigated. Further work is needed to determine the specific mechanisms behind each significant association found and more work is needed to understand the drivers behind the extremely high CS rates.

## Data Availability

The datasets used and/or analysed during the current study are available from the corresponding author on reasonable request.

## Abbreviations

NCDs: Non-communicable diseases
CS: Caesarean sections
PTB: Preterm birth
LMICs: Low middle Income countries
cOR: Crude odds ratio
aOR: Adjusted odds ratio
